# Genomic, antigenic, and seroepidemiological characterization of an antigenically divergent Tibet orbivirus strain isolated from *Culicoides* in China

**DOI:** 10.3389/fmicb.2026.1867318

**Published:** 2026-09-03

**Authors:** Yiling Yang, Yantao Zhu, Yuwen He, Zhenxing Yang, Keping Zhao, Qiang Sun, Yiju Chen, Jing Wu, Hong Liu, Zhao Li, Liming Zhang, Jianzhong Yin, Jinglin Wang

**Affiliations:** 1 Yunnan Key Laboratory of Cross-Border Infectious Disease Control and Novel Drug Development (Under Construction), School of Public Health, Kunming Medical University, Kunming, China; 2 Yunnan Tropical and Subtropical Animal Viral Disease Laboratory, Key Laboratory of Transboundary Animal Diseases Prevention and Control (Coconstruction by Ministry and Province), Yunnan Animal Science and Veterinary Institute, Kunming, China; 3 Department of Medical Genetics, Institute of Medical Biology, Chinese Academy of Medical Sciences and Peking Union Medical College, Kunming, China; 4 Mangshi Animal Disease Prevention and Control Center, Mangshi, Yunnan, China; 5 Shandong Provincial Research Center for Bioinformatic Engineering and Technique, School of Life Sciences and Medicine, Shandong University of Technology, Zibo, China; 6 Jiangcheng County Animal Disease Prevention and Control Center, Jiangcheng, Yunnan, China; 7 Tsinghua-Peking Center for Life Sciences, School of Medicine, Tsinghua University, Beijing, China

**Keywords:** *Culicoides* spp., livestock, microneutralization, putative novel serotype, Tibet orbivirus

## Abstract

Tibet orbivirus (TIBOV) is a poorly characterized orbivirus whose antigenic diversity and circulation in livestock remain incompletely defined. Here, we report the isolation of a TIBOV strain, MS18C217-16, from *Culicoides* spp. collected in Mangshi, Yunnan Province, China, in 2018. MS18C217-16 replicated efficiently in multiple mammalian cell lines and induced clear cytopathic effects. Phylogenetic analysis assigned the isolate to the TIBOV clade, with high amino acid conservation in VP1, VP3, and VP7, but marked divergence in the outer-capsid proteins VP2 and VP5. Structural modeling and predicted MHC class II-binding peptide profiles suggested distinctive antigenic features in these proteins, and neutralization assays provided evidence that MS18C217-16 is antigenically distinct from the reference TIBOV strain DH13C120. Serological surveys detected neutralizing antibodies in goats, swine, and cattle across four counties, with particularly high seropositivity in goats from Mengla. Together, these data identify MS18C217-16 as an antigenically divergent TIBOV strain with features consistent with a putative novel serotype, while highlighting the need for reciprocal cross-neutralization assays using strain-specific antisera for formal serotype confirmation.

## Introduction

Arboviruses are viral pathogens that are biologically transmitted between hematophagous arthropod vectors and susceptible vertebrate hosts and are responsible for a wide range of diseases in humans and animals (Weaver and Reisen, 2010). Members of the genus *Orbivirus*, family *Sedoreoviridae*, are non-enveloped, icosahedral viruses with genomes comprising 10 segments of double-stranded RNA that encode seven structural proteins (VP1–VP7) and at least four nonstructural proteins (NS1–NS4) (Ayllón et al., 2020). Orbiviruses are transmitted by several groups of hematophagous arthropods, including mosquitoes, ticks, and biting midges. Among them, *Culicoides*-borne orbiviruses, particularly bluetongue virus, epizootic hemorrhagic disease virus, and African horse sickness virus, are major veterinary pathogens because of their broad host ranges, capacity for geographic spread, and substantial effects on livestock and wildlife health (Mellor et al., 2000).

Tibet orbivirus (TIBOV) was first isolated as strain XZ0906 from *Anopheles maculatus* mosquitoes collected in Tibet, China, in 2009 and was subsequently characterized as a distinct member of the genus *Orbivirus* (Li et al., 2014). TIBOV strains were later isolated from mosquitoes and *Culicoides* biting midges in Yunnan and several other provinces of China (Lei et al., 2015; Wang et al., 2017). Two additional strains, KSB-8/C/09 and KSB-3/C/10, were isolated from *Culicoides* collected in Kagoshima, Japan, providing the first reported isolation of TIBOV outside China (Suda et al., 2021).

More recent genomic investigations have revealed substantial genetic diversity among TIBOV strains collected from different geographic regions, vector species, and vertebrate hosts (Li et al., 2022; Duan et al., 2023; Gao et al., 2024). Neutralizing antibodies against TIBOV have been detected in cattle, Asian buffaloes, goats, and swine, and at least one infectious strain has been recovered from sentinel cattle, supporting natural infection and silent circulation of TIBOV in livestock populations (Wang et al., 2017; Li et al., 2022). Nevertheless, the clinical significance, host range, and pathogenic potential of TIBOV remain incompletely understood.

Serotype classification is particularly important for orbiviruses because immunity is largely directed against serotype-specific outer-capsid antigens. Serotypes are conventionally distinguished using virus-neutralization assays, with the outer-capsid protein VP2, encoded by genome segment 2 and also designated outer capsid protein 1, serving as the principal neutralization antigen; VP5, encoded by segment 6, may additionally contribute to antigenic specificity (Matthijnssens et al., 2022). The marked divergence between the VP2 proteins of the prototype strain XZ0906 and the Chinese strain DH13C120 initially suggested that these viruses represented distinct TIBOV serotypes (Wang et al., 2017).

Subsequently, genomic analysis of the Japanese isolates KSB-8/C/09 and KSB-3/C/10 identified extensive Seg-2/VP2 divergence and proposed that these isolates represented two additional putative serotypes, resulting in a provisional four-serotype classification comprising TIBOV-1 to TIBOV-4 (Suda et al., 2021). Later studies expanded this scheme to six putative serotypes, predominantly on the basis of VP2 sequence divergence and phylogenetic clustering, with supporting evidence from VP5 analyses and serological testing of selected strains (Li et al., 2022; Duan et al., 2023). However, the nomenclature has not been fully harmonized because different isolates have been assigned the designations TIBOV-5 and TIBOV-6 in independent studies. Moreover, comprehensive reciprocal cross-neutralization assays involving representative strains of all proposed serotypes have not yet been reported. Therefore, the currently described TIBOV serotypes should be regarded as provisional or putative rather than as a fully standardized serological classification.

Here, we characterized the TIBOV isolate MS18C217-16, recovered from *Culicoides* biting midges collected in Mangshi, Yunnan Province, China, in 2018. We combined whole-genome sequencing, phylogenetic analyses of the outer-capsid proteins, structural modeling, antigenic characterization, and seroepidemiological investigations to determine whether MS18C217-16 represents an antigenically distinct TIBOV lineage and a candidate previously unrecognized putative serotype. We further evaluated its replication kinetics in cultured cells and investigated evidence of its circulation among local livestock. These findings expand current knowledge of the genetic and antigenic diversity of TIBOV and provide a foundation for improved surveillance, serotype classification, and risk assessment of this emerging orbivirus.

## Materials and methods

### Midge collection and processing

In August 2018, *Culicoides* midges were collected using light traps at a pig farm in Mangshi, Dehong Prefecture, Yunnan Province, China. Captured midges were immobilized by freezing at −20 °C for 20 min and were subsequently identified morphologically under a stereomicroscope as *Culicoides* spp. Approximately 100 midges were pooled per tube and stored in liquid nitrogen until further processing.

### Virus isolation

Cryopreserved *Culicoides* specimens were homogenized, and 200 μL of clarified supernatant was inoculated onto confluent monolayers of *Aedes albopictus* C6/36 cells. The cultures were monitored daily for cytopathic effects (CPE) for 7 days. Supernatants from cultures showing CPE were subjected to three blind passages in C6/36 cells. Virus-containing supernatants from the third passage were harvested and stored at −80 °C.

### Viral RNA extraction and full-length genome amplification

Total RNA was extracted from CPE-positive culture supernatants using TRIzol Reagent (TaKaRa) according to the manufacturer's instructions. Viral dsRNA was examined by electrophoresis on a 10% discontinuous polyacrylamide gel in Tris-glycine buffer, followed by silver staining. Full-length genome amplification was performed using the full-length amplification of cDNAs (FLAC) method as previously described (Ren et al., 2021). Briefly, dsRNA was ligated using T4 RNA ligase (New England Biolabs), reverse-transcribed with PrimeScript II Reverse Transcriptase (TaKaRa), and amplified by PCR. PCR products were cloned into the pMD19-T vector (TaKaRa), transformed into *Escherichia coli* DH5α competent cells (TaKaRa), and sequenced.

### Sequence assembly and phylogenetic analysis

Raw sequence reads were assembled using SeqMan in the DNASTAR Lasergene package (DNASTAR, Inc., Madison, WI, USA). Open reading frames were predicted using EditSeq (DNASTAR, Inc., Madison, WI, USA). Nucleotide and deduced amino acid sequence identities were analyzed using Clustal X (University College Dublin, Dublin, Ireland) and MegAlign (DNASTAR, Inc., Madison, WI, USA), and heatmaps were generated using TBtools, (South China Agricultural University, Guangzhou, Guangdong, China) (Chen et al., 2020). Representative orbivirus sequences were retrieved from GenBank for comparative analysis. Maximum-likelihood phylogenetic trees were reconstructed in MEGA-X using the best-fit substitution model and 1,000 bootstrap replicates (Kumar et al., 2018).

### Time-scaled phylogenetic and population dynamic analyses

Following the analytical framework described by Gao et al. (2024), time-scaled phylogenetic analysis was performed using segment 10 coding sequences from representative TIBOV strains. Segment 10 was selected because it provided the most complete sequence coverage among the available TIBOV strains. Sequences were aligned using MAFFT v7.505.

Potential recombination signals were assessed using the Phi test implemented in SplitsTree v6.7.9 (University of Tübingen, Tübingen, Germany). Substitution saturation was evaluated using Xia's test implemented in DAMBE v5.3.48 (University of Ottawa, Ottawa, ON, Canada) to determine whether the sequence dataset retained sufficient phylogenetic information. The HKY+F+G4 nucleotide substitution model was selected using ModelFinder in PhyloSuite v1.2.2 (Institute of Hydrobiology, Chinese Academy of Sciences, Wuhan, China) according to the Bayesian information criterion (Kalyaanamoorthy et al., 2017; Zhang et al., 2020). Combinations of molecular clock models and coalescent priors were compared using marginal likelihood estimates and Bayes factors. An uncorrelated lognormal relaxed molecular clock combined with a Bayesian skyline coalescent prior was identified as the best-supported model combination. Bayesian Markov chain Monte Carlo analyses were performed in BEAST v1.10.4 (BEAST Developers, University of Auckland, Auckland, New Zealand; University of Edinburgh, Edinburgh, UK; University of California, Los Angeles, CA, USA) for 1 × 10^8^ generations, with parameters sampled every 10,000 generations (Suchard et al., 2018). The first 10% of sampled states were discarded as burn-in. Convergence and mixing were assessed using Tracer v1.7.1 (University of Edinburgh, Edinburgh, UK), with effective sample size values greater than 200 considered indicative of adequate sampling. A maximum clade credibility tree was generated using TreeAnnotator v1.10.4 [BEAST Developers (University of Edinburgh, Edinburgh, UK and University of Auckland, Auckland, New Zealand)] after discarding the first 10% of trees as burn-in, with node heights summarized as median estimates. The resulting tree was visualized using FigTree v1.4.3 (University of Edinburgh, Edinburgh, UK) . Changes in relative genetic diversity through time were reconstructed using the Bayesian skyline model (Rambaut et al., 2018).

### Protein structural modeling and MHC class II-binding peptide prediction

The VP2 and VP5 amino acid sequences of MS18C217-16 and seven comparative TIBOV strains (DH13C120, XZ0906, KSB-3/C/10, P110, YNV/KM-1, YNV/17-14, and KSB-8/C/09) were subjected to structural analysis. Homology models were generated using SWISS-MODEL with PDB entries 8w1o.1.L for VP2 and 3j9e.1.A for VP5 as templates (Waterhouse et al., 2018). Structural visualization and surface electrostatic potential analyses were performed in VMD (Humphrey et al., 1996). For MHC class II-binding peptide analysis, MS18C217-16 was compared with one representative strain from each of the six proposed TIBOV serotypes: XZ0906 (TIBOV-1), DH13C120 (TIBOV-2), KSB-8/C/09 (TIBOV-3), KSB-3/C/10 (TIBOV-4), YNV/KM-1 (TIBOV-5), and YNV/17-14 (TIBOV-6). Putative MHC class II-binding peptides derived from VP2 and VP5 were predicted using NetMHCIIpan 4.0 (Reynisson et al., 2020) for four SLA class II alleles reported to be prevalent in Chinese swine: SLA-DQB1^*^0501, SLA-DRB1^*^090101, SLA-DRB1^*^0701, and SLA-DRB1^*^0501 (Belaganahalli et al., 2015). Predicted 15-mer peptides with binding affinities of < 500 nM were classified as high-affinity binders.

For each protein, predicted high-affinity peptide sequences were pooled across the four SLA class II alleles and deduplicated within each strain. MS18C217-16 was then compared pairwise with each of the six serotype representatives. For each comparison, predicted peptide repertoires were classified into peptides unique to MS18C217-16, peptides shared by both strains, and peptides unique to the corresponding serotype representative. Pairwise peptide-repertoire similarity was quantified using the Jaccard similarity index, calculated as the number of shared peptide sequences divided by the total number of unique peptide sequences in the union of the two peptide sets.

### Virus growth kinetics

BHK-21 cells were kindly provided by Professor Gong Cheng (Tsinghua University, Beijing, China). Vero, PK-15, HeLa, MDBK, and C6/36 cells were obtained from the cell repository of the Yunnan Tropical and Subtropical Animal Viral Disease Laboratory, Key Laboratory of Transboundary Animal Diseases Prevention and Control (Kunming, China). The replication kinetics of MS18C217-16 were compared with those of the reference strain DH13C120 in BHK-21, Vero, PK-15, HeLa, MDBK, and C6/36 cells. DH13C120 was isolated from *Culicoides* collected in Dehong in 2013, identified as TIBOV by molecular methods, and preserved in our laboratory (Wang et al., 2017). Cells were infected at a multiplicity of infection (MOI) of 0.01. Culture supernatants were collected every 12 h for 7 days, and viral titers were determined on BHK-21 cells by the 50% tissue culture infectious dose (TCID_50_) assay using the Reed–Muench method (Lei et al., 2021). For each virus–cell line combination, viral titers were measured in three parallel wells at each sampling time, and the mean of the three wells was used for curve visualization and statistical analysis.

### Statistical analysis of viral replication kinetics

Viral titers were expressed as log_10_ TCID50/mL and analyzed separately for each cell line using Gaussian generalized additive models with cubic regression splines. Because only the mean of three parallel wells was retained for each virus–time combination, the triplicate mean at each sampling time was treated as the observational unit, and no replicate-level random effect was included.

For each cell line, a full model allowing separate virus-specific temporal trajectories was compared with a reduced model containing a common temporal trajectory for MS18C217-16 and DH13C120 using a partial F test. Cubic B-splines with three basis degrees of freedom were used to limit overfitting across the eight sampling times. *P* values from the six cell-line analyses were adjusted using the Benjamini–Hochberg false-discovery-rate procedure. Adjusted *P* values below 0.05 were considered statistically significant. Model robustness was additionally assessed using spline basis dimensions ranging from three to five.

### Serological survey by microneutralization assay

A total of 680 serum samples from cattle, goats, and swine were collected in 2018 from four counties in Yunnan Province, China, including Mangshi, Mengla, Jinghong, and Jiangcheng. Serum samples were collected with informed consent from the animal owners. All sera were heat-inactivated at 56 °C for 30 min before testing. Two-fold serial dilutions of serum samples, starting at 1:10, were prepared in maintenance medium and dispensed into 96-well plates in triplicate (50 μL per well). An equal volume (50 μL) of virus suspension containing 100 TCID_50_ of MS18C217-16 was added to each well, and the plates were incubated at 37 °C for 1 h. Subsequently, 100 μL of BHK-21 cell suspension (1 × 10^5^ cells/mL) was added to each well. Plates were incubated and monitored daily for CPE for 5–7 days. Virus control, cell control, and negative serum control wells were included in each assay. Neutralizing antibody titers were determined as the highest serum dilution that inhibited virus-induced CPE in 50% of replicate wells, according to the Reed–Muench method. Samples with neutralizing antibody titers ≥1:10 were considered seropositive.

## Results

### Isolation of MS18C217-16

In 2018, a total of 8,500 *Culicoides* midges were collected from a pig farm in suburban Mangshi, Dehong Prefecture, Yunnan Province, and grouped into 85 pools for virus isolation in C6/36 cells. One isolate, designated MS18C217-16, produced evident cytopathic effects (CPE) in C6/36 cells at 96 h post-inoculation during the second passage. After three serial passages, CPE appeared reproducibly at 72 h post-inoculation and was characterized by increased refractility, cell rounding, and detachment.

### Polyacrylamide gel electrophoresis profile

**Figure 1:**
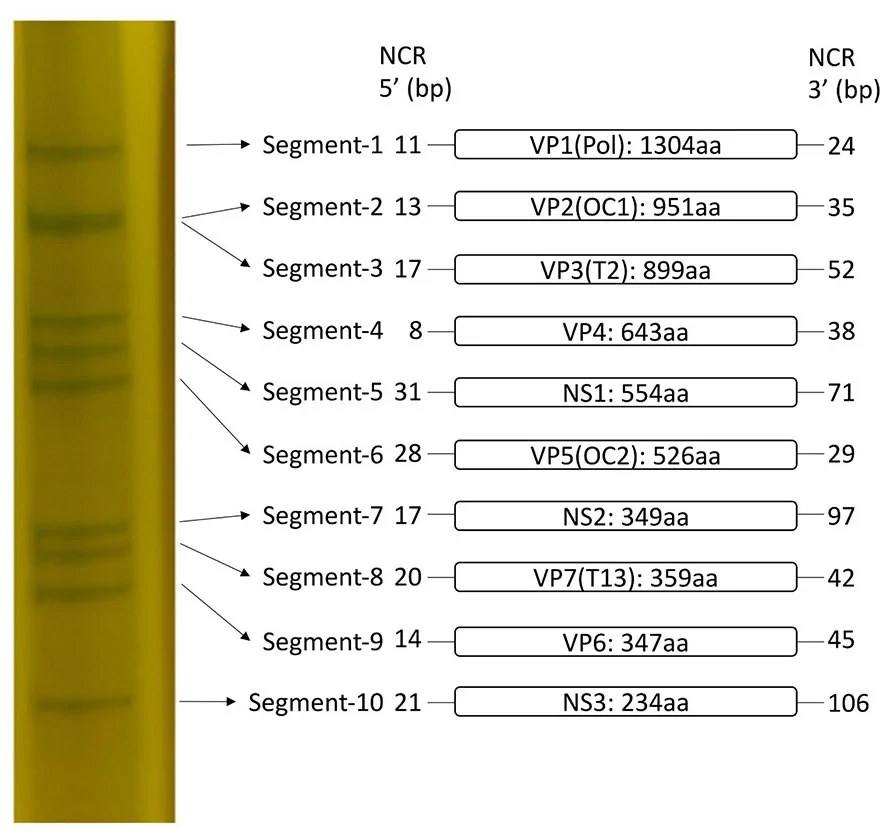
Polyacrylamide gel electrophoretic profile of the 10-segmented dsRNA genome of TIBOV strain MS18C217-16.

Polyacrylamide gel electrophoresis (PAGE) showed that MS18C217-16 possessed a 10-segmented dsRNA genome with a typical 3-3-3-1 migration pattern (Figure 1). Most segments migrated separately, whereas Seg-2 and Seg-3 co-migrated in this gel system, yielding an apparent “1-2-1-1-1-1-1-1-1” electrophoretic profile.

### Genome organization and characteristics of MS18C217-16

The complete genome of MS18C217-16 was determined by FLAC and sequencing. The terminal sequences of all 10 genome segments were further examined because conserved termini are characteristic features of orbivirus genomes and are involved in genome replication and packaging. As shown in Table 1, all segments of MS18C217-16 shared a conserved 5′ terminal motif beginning with GUAAAA and a conserved 3′ terminal motif ending with ACUUAC. The terminal four nucleotides at the 5′ and 3′ ends were reverse complementary, consistent with the conserved terminal organization reported for TIBOV and other orbiviruses. These conserved terminal motifs, together with the 10-segmented dsRNA genome organization, further support the assignment of MS18C217-16 to Tibet orbivirus (Table 1). The sequences of all segments were deposited in GenBank under accession numbers PP358565-PP358574. Compared with the TIBOV reference strain DH13C120, MS18C217-16 contained a 3-bp insertion in the open reading frame (ORF) of Seg-2, an 8-bp deletion in the 3′ non-coding region (NCR) of Seg-5, a 3-bp deletion in the 5′ UTR and a 1-bp deletion in the 3′ UTR of Seg-6, a 1-bp deletion in the 3′ UTR of Seg-7, and a 3-bp insertion in the ORF of Seg-9. Conserved terminal hexanucleotides were identified at the 5′ and 3′ ends of the genome (5′-GUAAAA…ACUUAC-3′), and the terminal four nucleotides were reverse complementary. The combined 5′ and 3′ UTRs accounted for 3.74% of the total genome, and the overall G + C content was 42.78%, comparable to that of other *Culicoides*-borne orbiviruses.

**Table 1:** Genome segment lengths, encoded proteins, and 5′ and 3′ non-coding regions of TIBOV strain MS18C217-16.

Segment	Length (bp)	GC%	Protein (aa)	5^′^NCR (bp)	Terminal sequences at the 5^′^and 3^′^ends	3^′^NCR (bp)	Putative function
S1	3,950	41.1	1,304	11	GUAAAAUCA-AUACACUUAC	24	VP1: RNA-dependent RNA polymerase
S2	2,904	40.8	951	13	GUAAAAATC-UCACACUUAC	35	VP2: outer capsid protein; major determinant of serotype specificity; highly variable protein; contains neutralizing epitopes
S3	2,769	44.3	899	17	GUAAAAUUU-ACAAACUUAC	52	VP3 (T2): forms sub-core capsid layer
S4	1,978	42.8	643	8	GUAAAAACA-UUACACUUAC	38	VP4: capping enzyme; sub-core protein with methyltransferase type 1 and type 2 activities
S5	1,767	43.9	554	31	GUAAAAAAG-UUACACUUAC	71	NS1: TuP; forms cytoplasmic tubules of unknown function
S6	1,638	43.6	526	28	GUAAAAAAG-UUAAACUUAC	29	VP5: outer capsid protein
S7	1,164	45.5	349	17	GUAAAAAUU-UUACACUUAC	97	VP7 (T13): immunodominant major serogroup-specific antigen
S8	1,142	43.9	359	20	GUAAAAAAU-UUAAACUUAC	42	NS2: single-stranded RNA-binding protein
S9	1,103	44.7	347	14	GUAAAAAAU-UAAAACUUAC	45	VP6: helicase for unwinding and binding to ssRNA and dsRNA; NTPase
S10	832	40.9	234	21	GUAAAAAAG-CUCGACUUAC	106	NS3: involved in cell exit; glycoprotein

In the “terminal sequence” column, sequences before and after the hyphen represent the 5′-terminal and 3′-terminal sequences of each genome segment, respectively, shown in the positive-sense orientation.

### Genome-wide sequence identity analysis

Comparative analyses of nucleotide and amino acid sequences across all 10 segments of MS18C217-16 and 12 comparative TIBOV strains revealed marked segment-specific variation, with the greatest divergence observed in the outer-capsid proteins VP2 and VP5 (Supplementary Figure S1). Among the structural proteins of MS18C217-16, VP1, VP3, VP4, VP6, and VP7 showed high amino acid identities to those of other TIBOV strains, ranging from 98.0% to 98.9%, 93.9% to 97.7%, 98.4% to 99.2%, 87.9% to 97.4%, and 92.8% to 99.7%, respectively. In contrast, VP2 and VP5 were substantially more variable, with amino acid identities ranging from 27.8% to 50.3% and from 64.0% to 78.9%, respectively.

At the nucleotide level, segment 2 of MS18C217-16 showed only 41.6% identity to strain V290/YNSZ, whereas the corresponding amino acid identity was 27.8%. For segment 6, the nucleotide and amino acid identities between KSB-8/C/09 and YNV/17-14 were 61.6% and 61.3%, respectively. In contrast, the remaining genomic segments generally showed nucleotide and amino acid identities above 70%. Among the nonstructural proteins, NS1 of MS18C217-16 shared the highest amino acid identity (89.4%) with strains D181/2008, YN15-283-01, SX-2017a, P110, and KSB-3/C/10; NS2 showed the highest identity (97.2%) with KSB-8/C/09 and V298/JNJH; and NS3 showed the highest identity (98.7%) with D181/2008.

### Sequence-based taxonomic placement of MS18C217-16 within the TIBOV lineage

**Figure 2:**
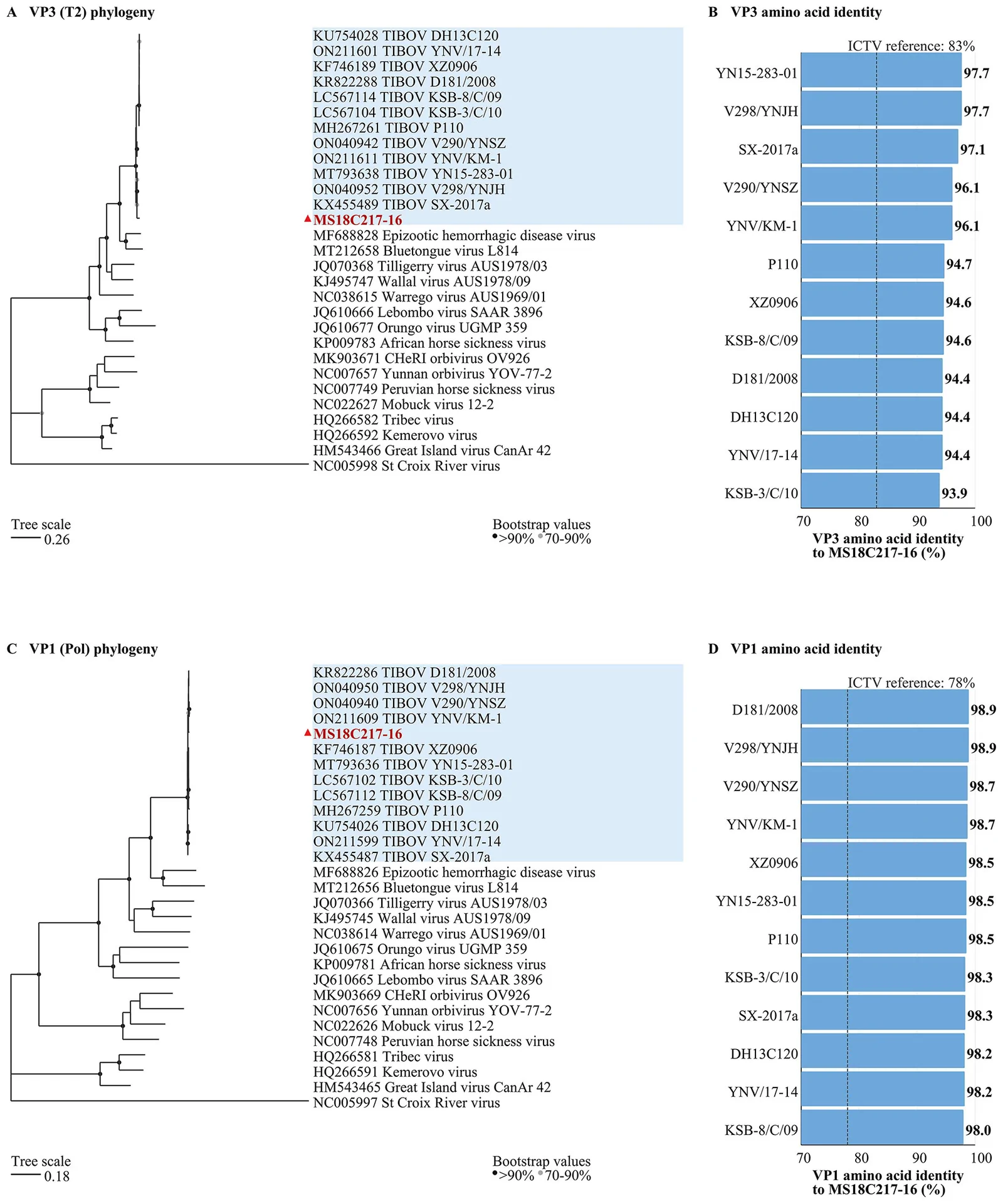
Sequence-based taxonomic placement of MS18C217-16 within the TIBOV lineage based on the conserved VP3 and VP1 proteins. **(A)** Maximum-likelihood phylogenetic tree based on VP3 (T2) amino acid sequences from MS18C217-16, representative TIBOV strains, and other orbiviruses. **(B)** Pairwise VP3 amino acid identities between MS18C217-16 and the examined TIBOV strains. The dashed vertical line indicates the 83% amino acid identity reference value normally observed among members of the same *Orbivirus* species according to the ICTV Report. **(C)** Maximum-likelihood phylogenetic tree based on VP1 (Pol) amino acid sequences. **(D)** Pairwise VP1 amino acid identities between MS18C217-16 and the examined TIBOV strains. The dashed vertical line indicates the corresponding 78% ICTV within-species reference value. In the phylogenetic trees, the TIBOV lineage is highlighted by light blue shading, and MS18C217-16 is indicated by a red triangle and red text. Filled circles indicate bootstrap support >90%, open circles indicate support of 70%−90%, and support values < 70% are not shown. Branch lengths are proportional to the number of amino acid substitutions per site. St. Croix River virus was used as the outgroup.

To establish the taxonomic position of MS18C217-16 before evaluating outer-capsid divergence, maximum-likelihood phylogenetic analyses were performed using the conserved VP3 (T2) and VP1 (Pol) proteins. In both phylogenies, MS18C217-16 clustered consistently within the TIBOV lineage and was clearly separated from representatives of other orbiviruses (Figures 2A, C).

According to the ICTV Report, members of the same *Orbivirus* species normally show >83% amino acid identity in the conserved VP3 protein and >78% amino acid identity in VP1 (Ayllón et al., 2020). VP3 of MS18C217-16 shared 93.9%−97.7% amino acid identity with the examined TIBOV strains (Figure 2B). The highest identities were observed with YN15-283-01 and V298/YNJH (97.7%), whereas the lowest identity was observed with KSB-3/C/10 (93.9%). Thus, all pairwise VP3 identities exceeded the corresponding ICTV within-species reference value.

Consistent with the VP3 analysis, VP1 was highly conserved between MS18C217-16 and the examined TIBOV strains, with amino acid identities ranging from 98.0 to 98.9% (Figure 2D). The highest identities were observed with D181/2008 and V298/YNJH (98.9%), whereas the lowest identity was observed with KSB-8/C/09 (98.0%). All VP1 identities substantially exceeded the corresponding ICTV within-species reference value. Together, the concordant phylogenetic placement and high sequence conservation of VP3 and VP1 support the identification of MS18C217-16 as a member of the TIBOV lineage.

### Outer-capsid divergence from the six proposed TIBOV serotypes

**Figure 3:**
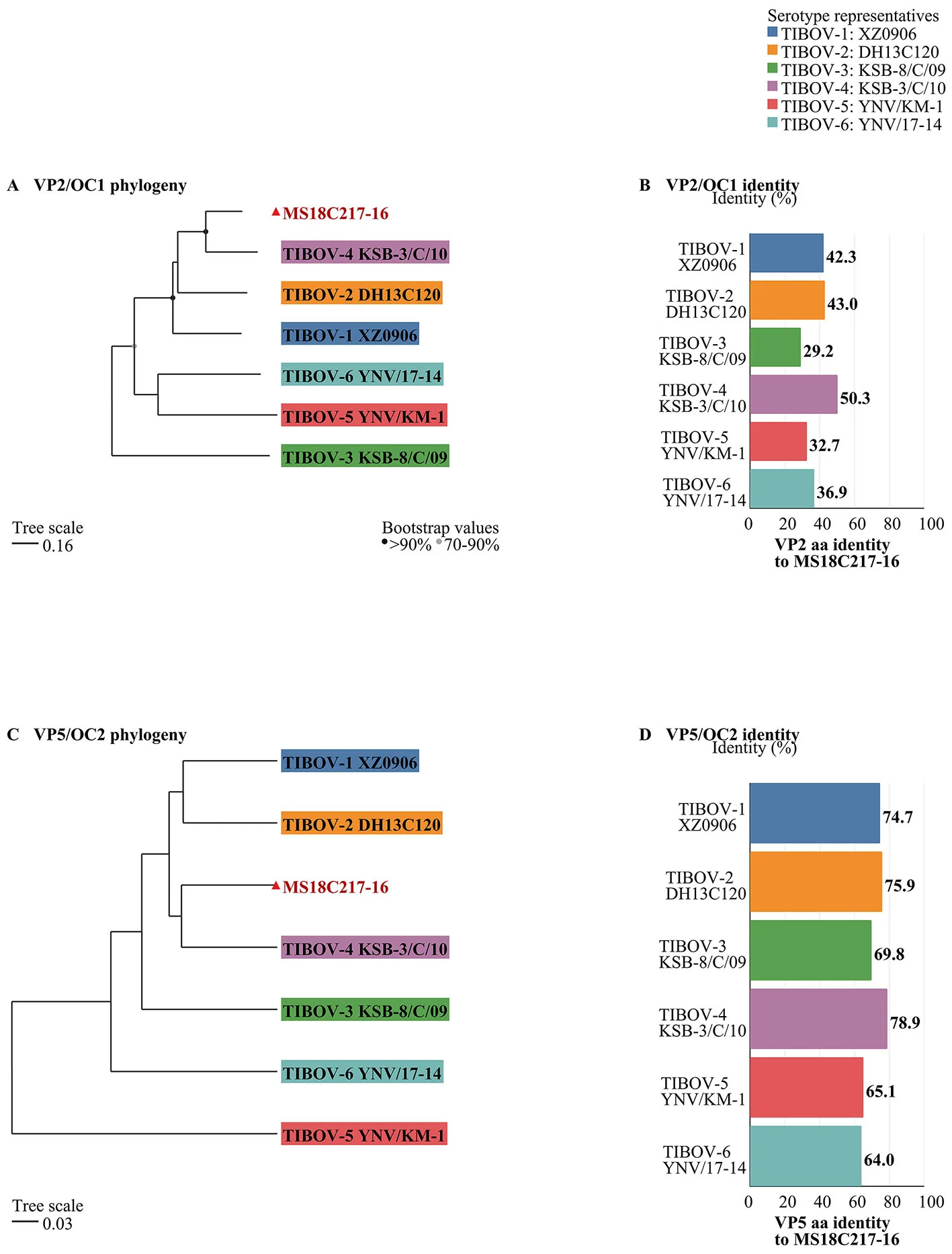
Outer-capsid divergence between MS18C217-16 and representatives of the six proposed TIBOV serotypes. **(A)** Maximum-likelihood phylogenetic tree based on VP2 (OC1) amino acid sequences from MS18C217-16 and one representative strain from each of the six proposed TIBOV serotypes. **(B)** Pairwise VP2 amino acid identities between MS18C217-16 and the six serotype representatives. **(C)** Maximum-likelihood phylogenetic tree based on VP5 (OC2) amino acid sequences from MS18C217-16 and the same six serotype representatives. **(D)** Pairwise VP5 amino acid identities between MS18C217-16 and the six serotype representatives. Representative strains were XZ0906 (TIBOV-1), DH13C120 (TIBOV-2), KSB-8/C/09 (TIBOV-3), KSB-3/C/10 (TIBOV-4), YNV/KM-1 (TIBOV-5), and YNV/17-14 (TIBOV-6). The same color scheme is used consistently for each proposed serotype across all panels. MS18C217-16 is indicated by a red triangle. Filled circles indicate bootstrap support >90%, open circles indicate support of 70%−90%, and support values < 70% are not shown. Branch lengths are proportional to the number of amino acid substitutions per site.

Having established the placement of MS18C217-16 within the TIBOV lineage, the serotype-associated outer-capsid proteins VP2 (OC1) and VP5 (OC2) were compared with one representative strain from each of the six proposed TIBOV serotypes (Figure 3).

VP2 phylogenetic analysis placed MS18C217-16 on a distinct branch relative to the six serotype representatives (Figure 3A). Pairwise amino acid identities were uniformly low, ranging from 29.2 to 50.3% (Figure 3B). The highest identity was observed with the TIBOV-4 representative KSB-3/C/10 (50.3%), followed by TIBOV-2 strain DH13C120 (43.0%), TIBOV-1 strain XZ0906 (42.3%), TIBOV-6 strain YNV/17-14 (36.9%), TIBOV-5 strain YNV/KM-1 (32.7%), and TIBOV-3 strain KSB-8/C/09 (29.2%). Thus, even the closest proposed serotype representative shared only approximately half of the VP2 amino acid sequence with MS18C217-16.

VP5 was more conserved than VP2 but remained substantially divergent across the six proposed serotypes. Pairwise amino acid identities ranged from 64.0 to 78.9% (Figure 3D). The highest identity was again observed with TIBOV-4 strain KSB-3/C/10 (78.9%), followed by TIBOV-2 strain DH13C120 (75.9%), TIBOV-1 strain XZ0906 (74.7%), TIBOV-3 strain KSB-8/C/09 (69.8%), TIBOV-5 strain YNV/KM-1 (65.1%), and TIBOV-6 strain YNV/17-14 (64.0%). Consistent with the sequence-identity analysis, the VP5 phylogeny placed MS18C217-16 closest to the TIBOV-4 representative while maintaining a distinct branch from all six proposed serotype representatives (Figure 3C).

Collectively, the VP2 and VP5 analyses demonstrate substantial outer-capsid divergence between MS18C217-16 and representatives of all six proposed TIBOV serotypes, with markedly greater divergence in VP2. Although TIBOV-4 was the closest proposed serotype in analyses of both outer-capsid proteins, the sequence divergence remained substantial, particularly for VP2. These findings provide a genomic basis for further evaluation of the antigenic position of MS18C217-16 but do not, by themselves, establish a distinct serotype.

### Time-scaled phylogeny and population dynamics

Before time-scaled phylogenetic reconstruction, the suitability of the segment 10 dataset was evaluated. The PHI test detected no statistically significant evidence of recombination (*P* = 0.76), indicating that the dataset was appropriate for phylogenetic inference. Xia's test showed that the observed substitution saturation index (ISS = 0.1987) was significantly lower than the corresponding critical value (ISS.C = 0.512; *P* < 0.0001), suggesting that the dataset retained sufficient phylogenetic signal for downstream analyses.

The HKY+F+G4 nucleotide substitution model was selected as the best-fitting model. An uncorrelated lognormal relaxed molecular clock combined with a Bayesian skyline coalescent prior was identified as the best-supported model combination. All key parameters exhibited effective sample size (ESS) values >200, indicating adequate convergence and mixing of the Markov chain Monte Carlo sampling.

**Figure 4:**
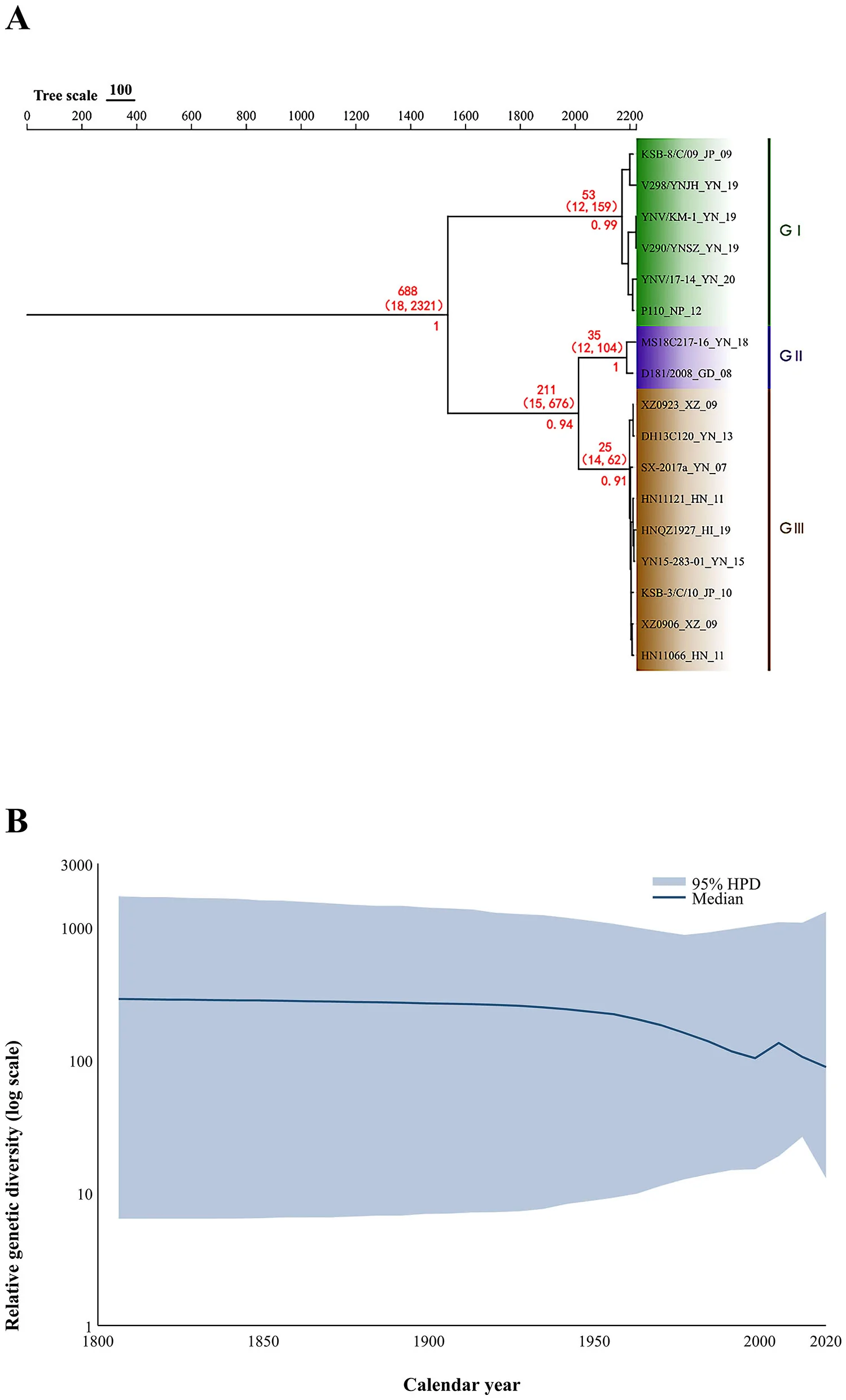
Time-scaled phylogeny and population dynamics of TIBOV based on segment 10 sequences. **(A)** Maximum clade credibility tree inferred under an uncorrelated lognormal relaxed molecular clock and a Bayesian skyline coalescent prior. The horizontal axis indicates time before present in years. Genotypes I–III are indicated by colored backgrounds. Selected internal nodes are annotated in red with median divergence-time estimates, with 95% highest posterior density (HPD) intervals shown in parentheses; posterior probabilities are shown below the corresponding nodes. **(B)** Bayesian skyline reconstruction of inferred relative genetic diversity through time. The solid line represents the posterior median, and the shaded region represents the pointwise 95% HPD interval. The *y*-axis is shown on a logarithmic scale.

The maximum clade credibility (MCC) tree resolved the sampled TIBOV strains into three major evolutionary lineages, designated genotypes I, II, and III (Figure 4A). The time to the most recent common ancestor (tMRCA) of all sampled TIBOV strains was estimated at approximately 688 years ago (95% highest posterior density [HPD]: 18–2321 years). Genotypes I, II, and III were estimated to have diverged approximately 53 years ago (95% HPD: 12–159 years), 35 years ago (95% HPD: 12–104 years), and 25 years ago (95% HPD: 14–62 years), respectively. MS18C217-16 clustered within genotype II together with strain D181/2008, whereas the prototype strain XZ0906 clustered within genotype III.

The Bayesian skyline reconstruction suggested that the inferred relative genetic diversity of the sampled TIBOV population remained broadly stable from the 19th century to the mid-20th century, followed by a gradual decline toward the present, with a modest transient increase around the early 2000s (Figure 4B). However, the 95% HPD interval remained broad throughout the reconstructed period, indicating substantial uncertainty in the inferred demographic trajectory.

### Comparative predicted MHC class II-binding peptide profiles across six proposed TIBOV serotypes

**Figure 5:**
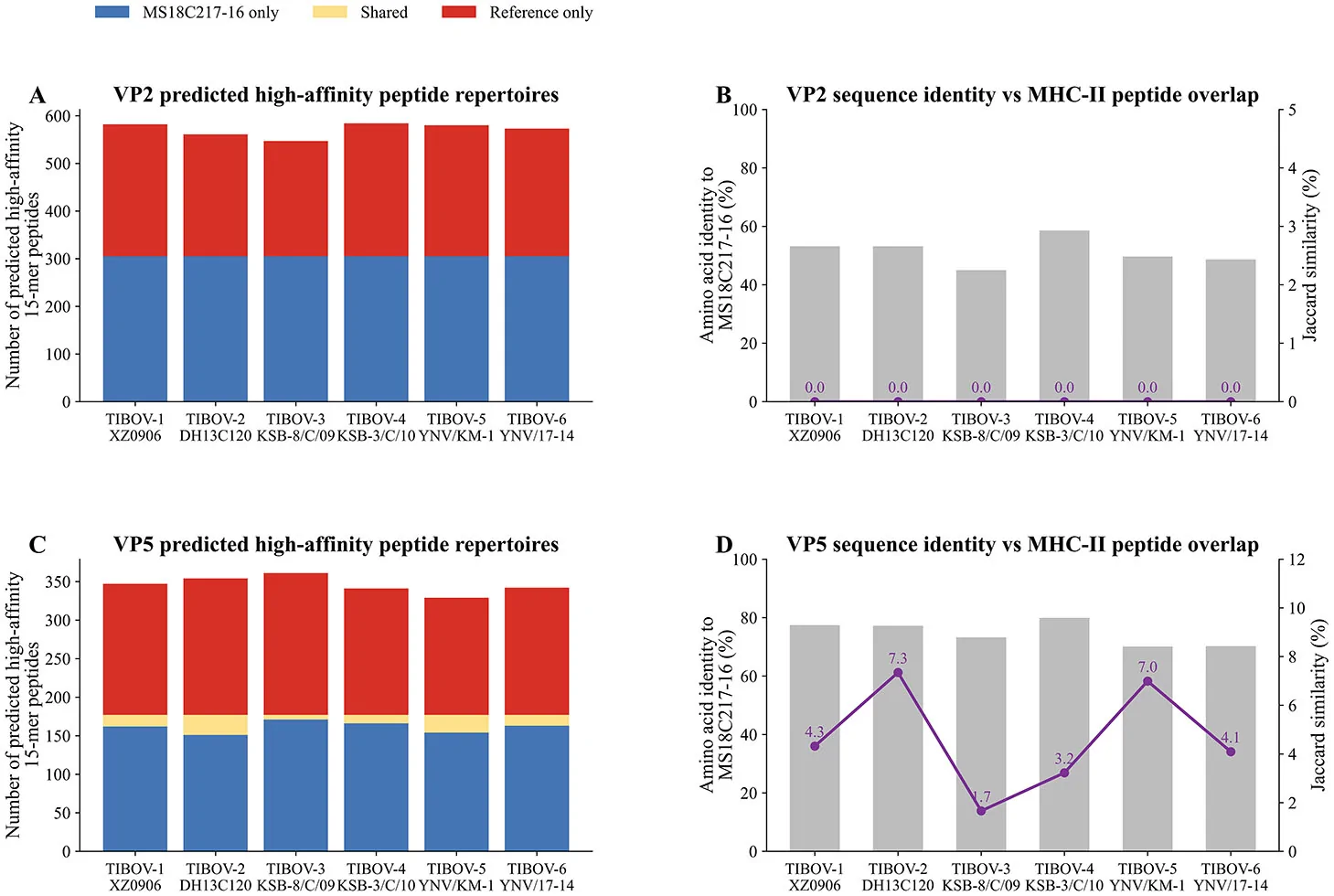
Predicted MHC class II-binding peptide profiles of VP2 and VP5 from TIBOV strain MS18C217-16 and representative strains of six putative TIBOV serotypes. Representative strains included XZ0906 (TIBOV-1), DH13C120 (TIBOV-2), KSB-8/C/09 (TIBOV-3), KSB-3/C/10 (TIBOV-4), YNV/KM-1 (TIBOV-5), and YNV/17-14 (TIBOV-6). Predicted high-affinity MHC class II-binding 15-mer peptides were identified using NetMHCIIpan 4.0 and were defined as peptides with predicted binding affinity < 500 nM. **(A, C)** Overlap of predicted high-affinity peptide repertoires between MS18C217-16 and each representative serotype strain for VP2 **(A)** and VP5 **(C)**. Blue indicates peptides unique to MS18C217-16, yellow indicates shared peptides, and red indicates peptides unique to the reference strain. **(B, D)** Amino acid identity to MS18C217-16 and Jaccard similarity of predicted high-affinity peptide repertoires for VP2 **(B)** and VP5 **(D)**. Gray bars indicate amino acid identity, and purple lines indicate strong-peptide Jaccard similarity. Although KSB-3/C/10 showed the highest VP2 and VP5 amino acid identity to MS18C217-16, its predicted MHC class II-binding peptide repertoire remained distinct from that of MS18C217-16.

To address the predicted MHC class II-binding peptide profiles across the proposed TIBOV serotype diversity, VP2 and VP5 of MS18C217-16 were compared with representative strains of the six proposed TIBOV serotypes, including XZ0906, DH13C120, KSB-8/C/09, KSB-3/C/10, YNV/KM-1, and YNV/17-14 (Figure 5). For VP2, MS18C217-16 contained 305 predicted high-affinity MHC class II-binding 15-mer peptides, whereas the six reference strains contained 242-279 peptides. No predicted high-affinity VP2 peptide was shared between MS18C217-16 and any of the six representative serotype strains. Notably, although KSB-3/C/10 showed the highest VP2 amino acid identity to MS18C217-16 among the reference strains, its predicted VP2 peptide repertoire remained completely distinct from that of MS18C217-16.

For VP5, MS18C217-16 contained 177 predicted high-affinity peptides, whereas the six reference strains contained 175–203 peptides. The number of shared VP5 peptides between MS18C217-16 and the reference strains was low, ranging from 6 to 26 peptides, corresponding to Jaccard similarity values of 1.7%−7.3%. KSB-3/C/10 again showed the highest VP5 amino acid identity to MS18C217-16, but shared only 11 predicted VP5 peptides with MS18C217-16. These results indicate that MS18C217-16 has a predicted MHC class II-binding peptide profile distinct from those of representative strains of the six proposed TIBOV serotypes, supporting predicted immunological divergence and providing a rationale for further serological evaluation.

### Predicted structural differences in VP2 and VP5

**Figure 6:**
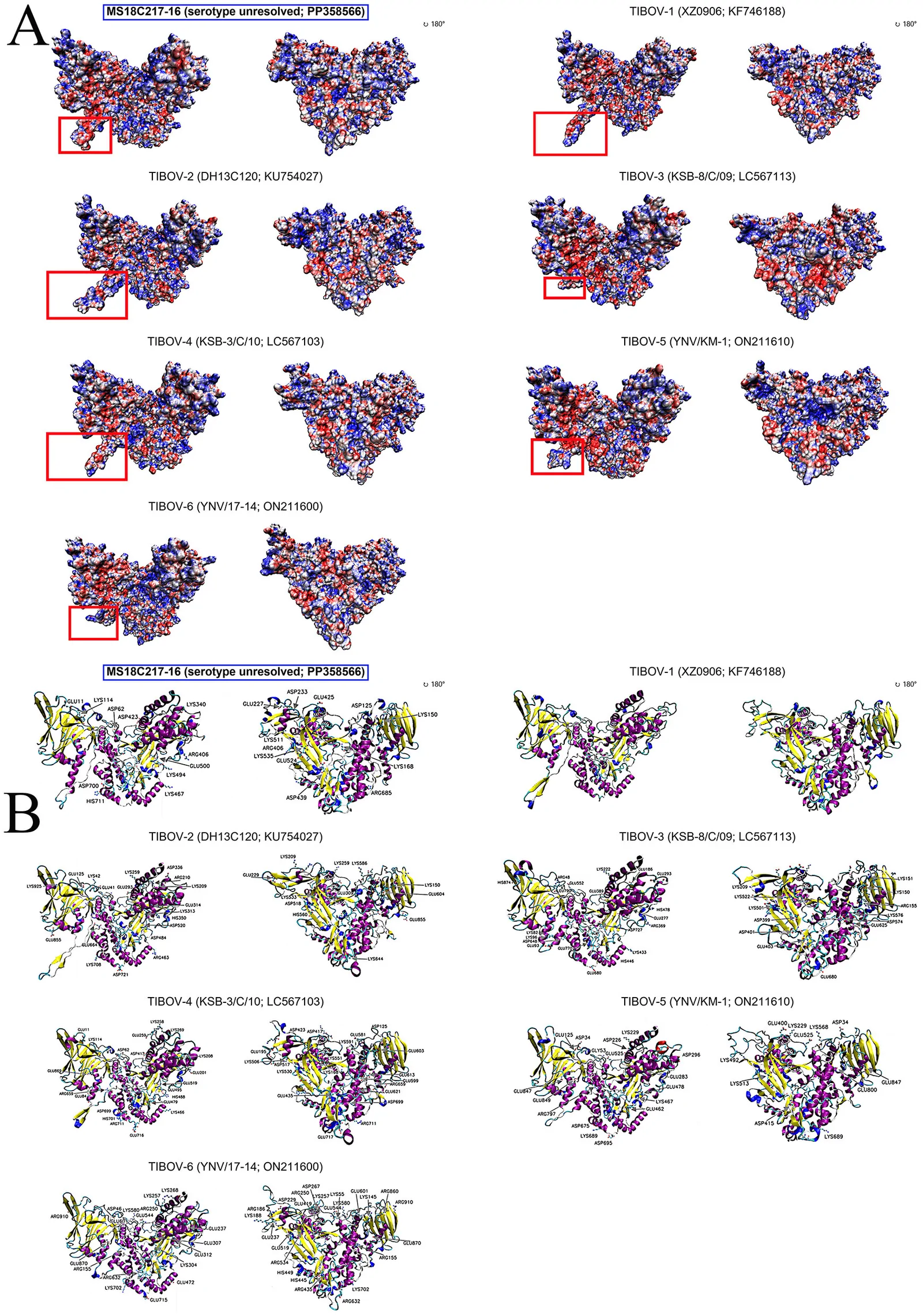
Structural model and surface electrostatic potential of the VP2 protein of TIBOV. **(A)** Surface electrostatic potential map of VP2. Blue indicates positive charge, red indicates negative charge, and white indicates neutral surface potential. The boxed region highlights the divergent domain identified in MS18C217-16. **(B)** Predicted three-dimensional structure of VP2, with variable residues highlighted.

**Figure 7:**
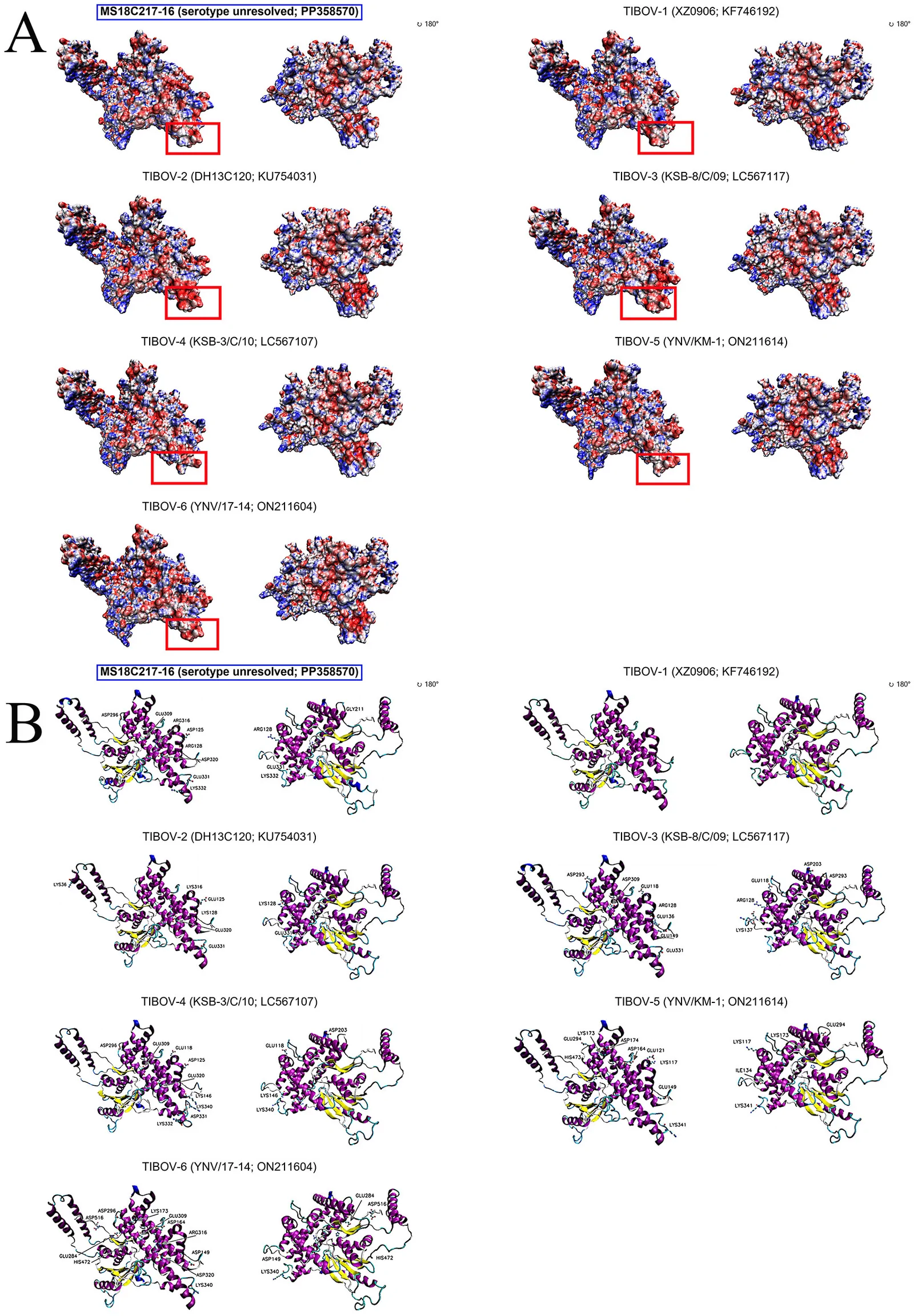
Structural model and surface electrostatic potential of the VP5 protein of TIBOV. **(A)** Surface electrostatic potential map of VP5. Blue indicates positive charge, red indicates negative charge, and white indicates neutral surface potential. The boxed region highlights the divergent domain identified in MS18C217-16. **(B)** Predicted three-dimensional structure of VP5, with variable residues highlighted.

Comparative structural modeling indicated that VP2 and VP5 of MS18C217-16 differed from those of the comparative TIBOV strains in predicted local conformation and surface electrostatic properties (Figures 6, 7). In VP2, the most pronounced differences were concentrated within a surface-exposed region spanning residues 80–100, where MS18C217-16 displayed distinct local conformation and charge distribution. In VP5, a region spanning residues 330–350 showed marked differences in predicted surface electrostatic properties relative to the comparison strains. Overall, the predicted structural differences were more extensive in VP2 than in VP5, consistent with the greater sequence divergence observed for VP2.

### Comparative microneutralization suggests antigenic divergence between MS18C217-16 and DH13C120

To further evaluate whether the marked divergence observed in VP2 and VP5 was associated with antigenic differences, comparative microneutralization assays were performed using field sera that were positive for TIBOV-neutralizing antibodies. Neutralization titers against MS18C217-16 were compared with those against DH13C120, a previously characterized TIBOV strain isolated from *Culicoides* in Yunnan Province.

The neutralization profiles differed between MS18C217-16 and DH13C120 for several serum samples (Table 2). Two-fold to four-fold differences in neutralization titers were observed in multiple sera. For example, serum DH70 neutralized MS18C217-16 at a titer of 1:40 but DH13C120 at 1:10, whereas serum ML27 neutralized MS18C217-16 at 1:160 but DH13C120 at 1:640. Similar differences were observed for additional goat and swine sera.

**Table 2:** Comparative microneutralization titers of field sera against MS18C217-16 and the TIBOV-2 strain DH13C120.

Serum source	Serum sample	Neutralizing titer to MS18C217-16	Neutralizing titer to DH13C120	Titer ratio (MS18C217-16/DH13C120)
Cattle	ML64	1:20	1:80	0.25
Goats	SZ41	1:20	1:10	2
	ML9	1:10	1:40	0.25
	DH70	1:40	1:10	4
Swine	ML7	1:320	1:160	2
	ML27	1:160	1:640	0.25
	ML37	1:40	1:160	0.25
	ML44	1:320	1:80	4
	JH1	1:40	1:20	2
	JH14	1:40	1:20	2
	JH36	1:40	1:80	0.5
	JH65	1:160	1:640	0.25
	JC139	1:160	1:40	4
	JC141	1:160	1:40	4

These findings indicate that MS18C217-16 is antigenically distinguishable from DH13C120. However, the observed differences were moderate and were based on field sera rather than strain-specific antisera. Moreover, the comparison included only DH13C120 and did not include reciprocal cross-neutralization against representative strains of all currently described TIBOV serotypes. Therefore, these data should be interpreted as supportive evidence of antigenic divergence from DH13C120, rather than definitive serological evidence for formal designation of MS18C217-16 as a novel TIBOV serotype.

### Replication kinetics in different cell lines

**Figure 8:**
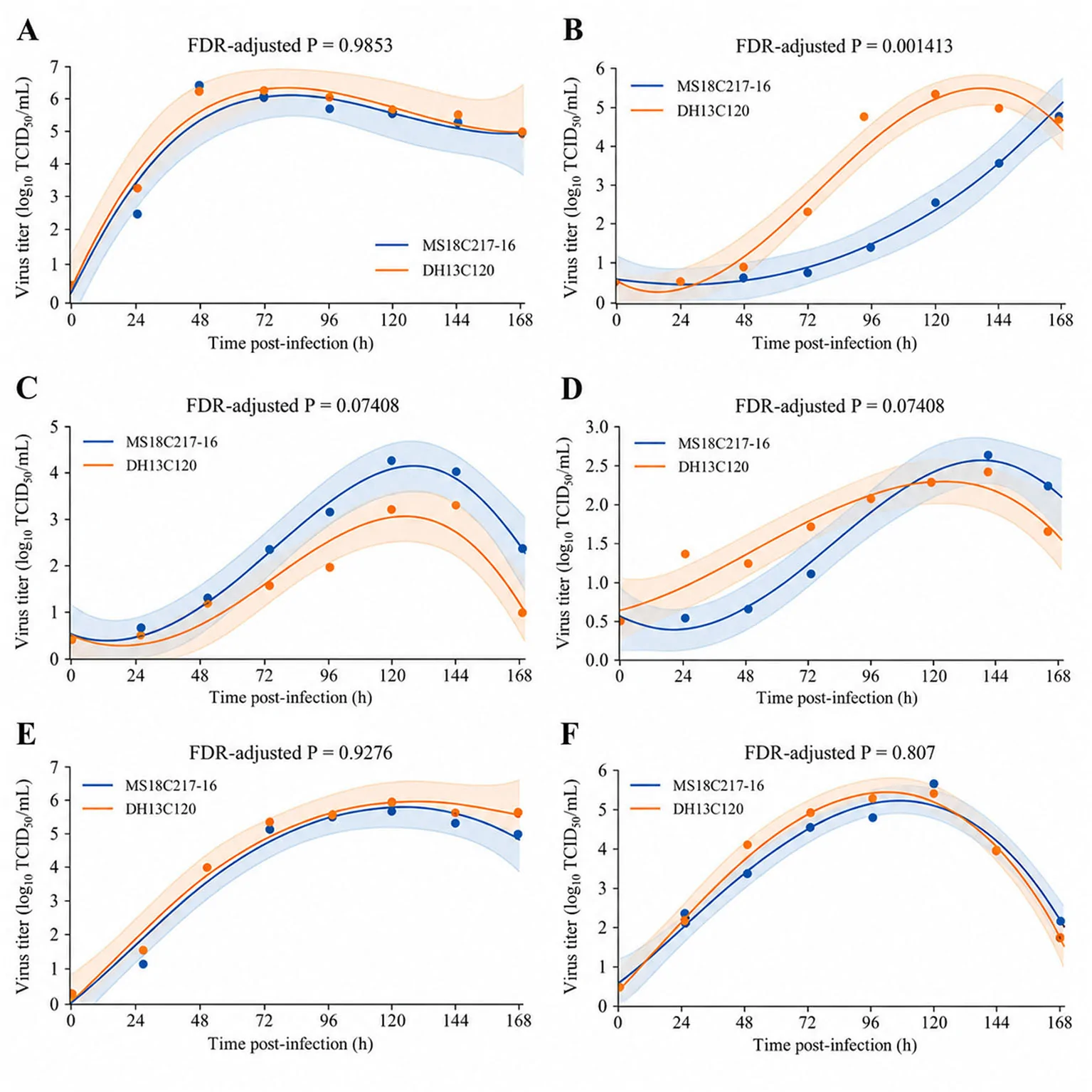
Comparative replication kinetics of TIBOV strains MS18C217-16 and DH13C120 in mammalian and insect cell lines. Viral replication was evaluated in BHK-21 **(A)**, C6/36 **(B)**, HeLa **(C)**, MDBK **(D)**, PK-15 **(E)**, and Vero **(F)** cells. Cells were infected at a multiplicity of infection of 0.01, and viral titers were determined at the indicated times post-infection and expressed as log_10_ TCID50/mL. At each time point, the titers from three parallel wells were averaged, and the resulting triplicate mean was used as the observational unit. Points represent the observed triplicate mean titers, solid lines represent virus-specific temporal trajectories fitted using Gaussian generalized additive models, and shaded areas indicate model-based 95% confidence intervals. For each cell line, a model allowing separate temporal trajectories for MS18C217-16 and DH13C120 was compared with a reduced model containing a common trajectory using a partial *F* test. *P* values were adjusted across the six cell-line comparisons using the Benjamini–Hochberg false-discovery-rate procedure, and the corresponding FDR-adjusted *P* values are shown in each panel.

The replication kinetics of MS18C217-16 and DH13C120 were compared in six cell lines, including BHK-21, Vero, PK-15, HeLa, MDBK, and C6/36 cells (Figure 8). Both strains replicated in all tested cell lines, although the magnitude and timing of peak titers differed among cell types. In BHK-21, Vero, and PK-15 cells, peak titers of both viruses exceeded 10^5.5^ TCID_50_/mL at approximately 2, 5, and 5 days post-inoculation, respectively, followed by variable declines. In HeLa and MDBK cells, both strains showed lower replication efficiency, with peak titers below 10^4.0^ TCID_50_/mL at approximately 5–6 and 4–6 days post-inoculation, respectively.

After false-discovery-rate correction, a statistically significant difference between the fitted temporal trajectories of MS18C217-16 and DH13C120 was detected only in C6/36 cells [*F*_(4, 8)_ = 21.726, raw *P* = 0.00024, FDR-adjusted *P* = 0.00141]. No statistically significant differences were detected in BHK-21, Vero, or PK-15 cells, whereas the nominal differences observed in HeLa and MDBK cells did not remain significant after FDR correction.

In C6/36 cells, MS18C217-16 showed a gradual increase in titer during the first 4 days, followed by a sharper rise thereafter, reaching its highest observed titer of 10^4.875^ TCID_50_/mL at 168 h post-infection. In contrast, DH13C120 increased more rapidly from 48 h, reached its highest observed titer of 10^5.25^ TCID_50_/mL at 120 h, and subsequently declined. These results demonstrate a statistically supported difference in the temporal replication trajectories of the two strains in C6/36 cells.

### Animal-level serological evidence of exposure to MS18C217-16 or antigenically related TIBOV variants

Serum samples from cattle, goats, and swine collected in Mengla, Mangshi, Jinghong, and Jiangcheng counties of Yunnan Province were tested for neutralizing antibodies against MS18C217-16 (Table 3). Neutralizing antibodies were detected in cattle, goats, and swine from Mengla and Mangshi, and in swine from Jinghong and Jiangcheng. The overall seropositivity rate was 12.79%. The highest seropositivity was observed in Mengla (23.86%), particularly in goats, for which the positivity rate reached 80.77%. Across the four regions, seropositivity ranked as follows: Mengla (23.86%), Jinghong (12.73%), Mangshi (8.11%), and Jiangcheng (7.41%). Among the three animal species, goats showed the highest seropositivity (38.46%), followed by swine (12.86%) and cattle (3.72%). No neutralizing antibodies were detected in cattle or goat serum samples from Jinghong or Jiangcheng (Table 3).

**Table 3:** Animal-level seropositivity of neutralizing antibodies against MS18C217-16 in livestock from Yunnan Province.

Livestock	Location (positive sample/total sample)				Positive rate in livestock (%)
	Mengla	Jinghong	Mangshi	Jiangcheng	
Cattle	10/100	0/42	1/89	0/65	3.72
Goats	21/26	0/18	19/40	0/20	38.46
Swine	11/50	14/50	1/130	10/50	12.86
Positive rate in location (%)	23.86	12.73	8.11	7.41	12.79

Seropositivity was defined as a neutralizing antibody titer ≥1:10.

## Discussion

Since Tibet orbivirus (TIBOV) was first isolated in 2009 from *Anopheles maculatus* collected in Motuo, Xizang, China, at least 18 TIBOV strains had been reported by 2020 from mosquitoes, *Culicoides* midges, and sentinel cattle in Yunnan, Hainan, Xizang, and Guangdong in China, as well as in Japan and Nepal (Gao et al., 2024; Li et al., 2025). Based primarily on nucleotide and amino acid sequence identities and phylogenetic analyses of the outer-capsid proteins VP2 and VP5, these strains have been provisionally assigned to multiple serotypes (Li et al., 2022). However, the host range, transmission ecology, evolutionary history, structural diversity, and pathogenic potential of newly recognized TIBOV variants remain incompletely understood. These knowledge gaps emphasize the need to characterize newly detected TIBOV isolates and to clarify their genetic, antigenic, and epidemiological properties.

In the present study, we isolated TIBOV strain MS18C217-16 from *Culicoides* midges collected in Yunnan Province, China, in 2018 and characterized its genomic, phylogenetic, antigenic, replication, and seroepidemiological features. Several characteristics supported its assignment to TIBOV, including the typical 3-3-3-1 genome migration pattern and conserved terminal nucleotide motifs in the 5′ and 3′ non-coding regions (5′-GUAAAA…ACUUAC-3′) (Li et al., 2022; Duan et al., 2023). In addition, the conserved VP3 and VP1 proteins showed high amino acid identities to those of previously reported TIBOV strains and consistently placed MS18C217-16 within the TIBOV lineage according to the ICTV sequence-based framework. Together, these genomic and phylogenetic findings support the classification of MS18C217-16 within Tibet orbivirus.

Within orbiviruses, the outer-capsid proteins VP2 and VP5 are among the most variable structural proteins and contribute substantially to antigenic diversity and serotype specificity (Hassan and Roy, 1999). In other orbivirus species, including bluetongue virus, epizootic hemorrhagic disease virus, and African horse sickness virus, VP2 sequence diversity is closely associated with serotype differentiation (Li et al., 2022). Consistent with this pattern, MS18C217-16 showed marked divergence from previously reported TIBOV strains, with only 29.2%−50.3% amino acid identity in VP2 and 64.0%−78.9% identity in VP5. Both proteins also occupied distinct phylogenetic positions relative to representative TIBOV strains. Structural comparisons and predicted MHC class II-binding peptide profiles further suggested differences in the potential antigenic and immunological properties of VP2 and VP5. These *in silico* findings should, however, be regarded as supportive evidence of predicted divergence rather than as direct evidence of serotype status.

Comparative neutralization using field sera revealed moderate differences in antibody titers against MS18C217-16 and DH13C120. These findings indicate that MS18C217-16 is antigenically distinguishable from DH13C120, but they do not establish a distinct serotype. Taken together, the marked VP2 and VP5 sequence divergence, distinct phylogenetic placement, structural differences, predicted peptide-profile differences, and differential neutralization relative to DH13C120 support the interpretation that MS18C217-16 is an antigenically divergent TIBOV strain with features consistent with a putative novel serotype. Nevertheless, formal serotype designation remains premature because reciprocal cross-neutralization assays using strain-specific antisera against MS18C217-16 and representative strains of all described TIBOV serotypes were not performed.

Vector competence remains one of the least understood aspects of TIBOV ecology. Among the strains reported to date, several have been isolated from mosquitoes, whereas others have been recovered from *Culicoides* midges (Gao et al., 2024). Experimental studies have shown that TIBOV can replicate in insect cell lines, including C6/36 and Aag2 cells (Ren et al., 2021), whereas experimental infection studies in *Aedes aegypti* and *Culex pipiens pallens* have suggested limited susceptibility in these mosquito species (Ren et al., 2024). In parallel, phylogenetic and genomic analyses have indicated that TIBOV is more closely related to *Culicoides*-borne orbiviruses than to mosquito-borne orbiviruses (Li et al., 2022). The isolation of MS18C217-16 from *Culicoides* midges provides additional evidence that biting midges may contribute to the natural transmission cycle of TIBOV. However, virus isolation from field-collected midges alone does not demonstrate vector competence. The specific *Culicoides* species involved and their ability to acquire, replicate, disseminate, and transmit TIBOV under experimental conditions remain to be determined.

Both MS18C217-16 and DH13C120 replicated in the tested mammalian and insect cell lines, although the magnitude and timing of viral replication varied among cell types. Generalized additive modeling provided robust statistical evidence that the temporal replication trajectories of the two strains differed in C6/36 cells [*F*_(4, 8)_ = 21.726, raw *P* = 0.00024, false-discovery-rate-adjusted *P* = 0.00141]. In this mosquito-derived cell line, DH13C120 increased earlier and reached its highest observed titer at 120 h, whereas MS18C217-16 showed a delayed increase and reached its highest observed titer at 168 h. In contrast, no statistically significant differences between the fitted temporal trajectories were detected in BHK-21, Vero, or PK-15 cells after correction for multiple testing. Nominal differences observed in HeLa and MDBK cells did not remain significant after false-discovery-rate correction. These findings support a strain-dependent difference in *in vitro* replication kinetics in C6/36 cells. However, cell-culture growth curves alone cannot establish differences in vector competence, animal host tropism, tissue tropism, or *in vivo* viral fitness.

Serological testing detected neutralizing antibodies against MS18C217-16 in livestock from multiple areas of Yunnan Province. The overall animal-level seropositivity was 12.79%, with the highest proportion observed in goats, followed by swine and cattle. Particularly high seropositivity was detected among the sampled goats from Mengla County. These findings provide evidence that livestock in the study areas had been exposed to MS18C217-16 or an antigenically related TIBOV variant. However, because the survey was cross-sectional and the distribution of sampled animals differed among species and geographic locations, the observed proportions should not be interpreted as unbiased estimates of regional population prevalence. The apparent differences among animal species and sampling locations may reflect variation in vector exposure, husbandry practices, sampling structure, or other unmeasured factors. Additional longitudinal and farm-structured surveys will be needed to determine the spatial distribution, force of infection, and host-specific exposure patterns of TIBOV.

The detection of neutralizing antibodies in cattle, goats, and swine suggests that TIBOV exposure is not restricted to a single livestock species. Nevertheless, seropositivity does not demonstrate clinical disease or pathogenicity. Although MS18C217-16 replicated in several mammalian and insect cell lines, its pathogenic potential has not been evaluated in experimentally infected animals. Further studies using controlled infection models, together with expanded vector and livestock surveillance, will be required to determine whether MS18C217-16 has veterinary or public-health significance.

## Limitations

This study has several limitations. First, although MS18C217-16 showed marked divergence in VP2 and VP5 and displayed moderate neutralization differences relative to DH13C120, formal reciprocal cross-neutralization assays using strain-specific antisera against MS18C217-16 and representative strains of all described TIBOV serotypes were not performed. In addition, the comparative neutralization assay used field sera rather than strain-specific antisera, and DH13C120 was the only reference strain included. Therefore, the neutralization results should be interpreted as evidence of antigenic divergence from DH13C120 rather than as definitive evidence of a novel serotype.

Second, the MHC class II-binding peptide analysis was based on *in silico* prediction and does not directly demonstrate antigen processing, T-cell recognition, or protective immunity. These predictions should therefore be considered hypothesis-generating and require experimental validation.

Third, the serological survey was cross-sectional and cannot determine the timing, duration, or direction of virus transmission. Differences in sample size and host composition among sampling locations may also have influenced the observed animal-level seropositivity estimates. Where complete farm-level metadata and herd sizes were unavailable, herd-level prevalence and farm-specific sampling fractions could not be reliably estimated.

Fourth, the growth-curve analysis was based on the mean titers of three parallel wells at each sampling time because individual well-level measurements were not retained. Consequently, the generalized additive models evaluated differences between summarized mean trajectories but could not estimate within-time-point variability or incorporate replicate-level random effects.

Fifth, the pathogenicity of MS18C217-16 was not evaluated in experimentally infected animals, and the clinical relevance of livestock exposure remains unknown. Finally, the *Culicoides* specimens were identified morphologically only to the genus level, and the specific vector species responsible for virus transmission remains undetermined.

## Conclusion and perspectives

In summary, MS18C217-16 is an antigenically divergent TIBOV strain characterized by substantial VP2 and VP5 sequence divergence, distinct outer-capsid phylogenetic placement, predicted structural and immunological differences, and moderate neutralization differences relative to DH13C120. These features are consistent with the possibility that MS18C217-16 represents a putative novel TIBOV serotype, although formal designation requires reciprocal cross-neutralization using strain-specific antisera against representative strains of all proposed TIBOV serotypes.

The detection of neutralizing antibodies in livestock from multiple regions of Yunnan provides evidence of exposure to MS18C217-16 or antigenically related TIBOV variants. In addition, the statistically supported difference in replication kinetics between MS18C217-16 and DH13C120 in C6/36 cells indicates that antigenically divergent TIBOV strains may also differ in their *in vitro* replication phenotypes. These findings expand current understanding of the genetic, antigenic, and biological diversity of TIBOV. Future studies should prioritize formal serological characterization, farm-structured and longitudinal surveillance, identification and experimental evaluation of competent vectors, and pathogenicity studies in susceptible animal hosts.

## Data Availability

The datasets presented in this study can be found in online repositories. The names of the repository/repositories and accession number(s) can be found in the article/Supplementary material.
