# Supplementary material for: Genomic, antigenic, and seroepidemiological characterization of an antigenically divergent Tibet orbivirus strain isolated from Culicoides in China
Source: Front Microbiol. 2026 Sep 3;17:1867318. doi: 10.3389/fmicb.2026.1867318 (PMC13582390; doi:10.3389/fmicb.2026.1867318)
Supplement: Supplementary file 1 [file Data_Sheet_1.docx]

**SUPPLEMENTARY MATERIALS**

**Supplementary Figure S1. Genome-wide sequence identity comparison of MS18C217-16 with representative TIBOV strains.**

(A) Pairwise amino acid identities of the proteins encoded by the 10 genome segments. (B) Pairwise nucleotide identities of the corresponding genome segments. Color gradients indicate the percentage of sequence identity between MS18C217-16 and representative TIBOV strains.

**Supplementary Figure S2. Supporting phylogenetic placement of MS18C217-16 based on VP7 (T13) amino acid sequences.** A maximum-likelihood phylogenetic tree was reconstructed using VP7 (T13) amino acid sequences from MS18C217-16, representative TIBOV strains, and other orbiviruses. The TIBOV lineage is highlighted by light-blue shading, and MS18C217-16 is indicated by a red triangle and red text. Bootstrap support was evaluated using 1,000 replicates. Filled circles indicate bootstrap support >90%, open circles indicate support of 70%–90%, and support values <70% are not shown. Branch lengths are proportional to the number of amino acid substitutions per site. St. Croix River virus was used as the outgroup.

**Supplementary Table S1. Core TIBOV strains and GenBank accession numbers used for serotype-level comparative analyses in this study.**

| **Strain** | **Proposed serotype used in this study** | **Country/region** | **Collection year** | **Isolation source** | **Complete genome GenBank accession numbers** | **Primary role in this study** | **Reference** |
| --- | --- | --- | --- | --- | --- | --- | --- |
| MS18C217-16 | Study strain; serotype status unresolved | Mangshi, Dehong, Yunnan, China | 2018 | *Culicoides* spp. pool collected at a pig farm | PP358565–PP358574 | Study strain | Present study |
| XZ0906 | TIBOV-1 | Motuo, Xizang, China | 2009 | *Anopheles maculatus* mosquitoes | KF746187–KF746196 | TIBOV-1 representative | Li et al., 2014 |
| DH13C120 | TIBOV-2 | Dehong, Yunnan, China | 2013 | *Culicoides* spp. | KU754026–KU754035 | TIBOV-2 representative; neutralization and growth-kinetics comparator | Wang et al., 2017 |
| KSB-8/C/09 | TIBOV-3 | Kagoshima, Japan | 2009 | *Culicoides* spp. | LC567112–LC567121 | TIBOV-3 representative | Suda et al., 2021 |
| KSB-3/C/10 | TIBOV-4 | Kagoshima, Japan | 2010 | *Culicoides* spp. | LC567102–LC567111 | TIBOV-4 representative | Suda et al., 2021 |
| YNV/KM-1 | TIBOV-5 | Yunnan, China | 2019ᵃ | *Culicoides* spp. / sentinel cattleᵇ | ON211609–ON211618 | TIBOV-5 representative | Li et al., 2022 |
| YNV/17-14 | TIBOV-6 | Yunnan, China | 2020ᵃ | *Culicoides* spp. / sentinel cattleᵇ | ON211599–ON211608 | TIBOV-6 representative | Li et al., 2022 |

**Notes:** Proposed serotype designations are those adopted in the present study and should be regarded as provisional because TIBOV serotype nomenclature has not been fully harmonized. GenBank accession ranges correspond to the 10 genome segments of each strain. MS18C217-16 is the study strain, and its formal serotype status remains unresolved pending reciprocal cross-neutralization assays using strain-specific antisera.
